# (*E*)-*N*′-(2-Hydroxy­benzyl­idene)-2-(4-isobutyl­phen­yl)propanohydrazide

**DOI:** 10.1107/S1600536809050971

**Published:** 2009-12-04

**Authors:** Jia Hao Goh, Hoong-Kun Fun, A. C. Vinayaka, B. Kalluraya

**Affiliations:** a X-ray Crystallography Unit, School of Physics, Universiti Sains Malaysia, 11800 USM, Penang, Malaysia; b Department of Studies in Chemistry, Mangalore University, Mangalagangotri, Mangalore 574 199, India

## Abstract

The title hydrazide compound, C_20_H_24_N_2_O_2_, exists in a *trans* configuration with respect to the acyclic C=N bond and an intra­molecular O—H⋯N hydrogen bond generates an *S*(6) ring motif. The mean plane through the formohydrazide unit is essentially planar [maximum deviation = 0.025 (2) Å], and forms dihedral angles of 24.45 (16) and 87.14 (16)° with the two benzene rings. In the crystal structure, inter­molecular N—H⋯O and C—H⋯O hydrogen bonds link neighbouring mol­ecules into extended chains along the *c* axis, which incorporate *R*_2_^2^(16) ring motifs. An inter­molecular C—H⋯π inter­action is also observed.

## Related literature

For the metal coordination and pharmacological activity of the title compound, see: Bedia *et al.* (2006 ▶); Rodrìguez-Argüelles *et al.* (2004 ▶); Rollas *et al.* (2002 ▶); Terzioglu & Gürsoy (2003 ▶). For hydrogen-bond motifs, see: Bernstein *et al.* (1995 ▶). For related structures, see: Fun *et al.* (2009**a* ▶,b* ▶). For bond-length data, see: Allen *et al.* (1987 ▶). For the stability of the temperature controller used for the data collection, see: Cosier & Glazer (1986 ▶).
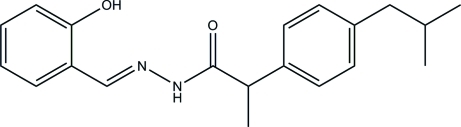


## Experimental

### 

#### Crystal data

C_20_H_24_N_2_O_2_
*M* *_r_* = 324.41
Monoclinic, 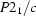

*a* = 5.5017 (2) Å
*b* = 33.0204 (14) Å
*c* = 9.7279 (4) Å
β = 91.731 (3)°
*V* = 1766.45 (12) Å^3^
*Z* = 4
Mo *K*α radiation
μ = 0.08 mm^−1^
*T* = 100 K
0.39 × 0.24 × 0.19 mm

#### Data collection

Bruker SMART APEXII CCD diffractometer
Absorption correction: multi-scan (*SADABS*; Bruker, 2005 ▶) *T*_min_ = 0.970, *T*_max_ = 0.985
18817 measured reflections
4059 independent reflections
3190 reflections with *I* > 2σ(*I*)
*R*_int_ = 0.042

#### Refinement

*R*[*F* ^2^ > 2σ(*F* ^2^)] = 0.076
*wR*(*F* ^2^) = 0.181
*S* = 1.18
4059 reflections
228 parameters
H atoms treated by a mixture of independent and constrained refinement
Δρ_max_ = 0.26 e Å^−3^
Δρ_min_ = −0.28 e Å^−3^

### 

Data collection: *APEX2* (Bruker, 2005 ▶); cell refinement: *SAINT* (Bruker, 2005 ▶); data reduction: *SAINT*; program(s) used to solve structure: *SHELXTL* (Sheldrick, 2008 ▶); program(s) used to refine structure: *SHELXTL*; molecular graphics: *SHELXTL*; software used to prepare material for publication: *SHELXTL* and *PLATON* (Spek, 2009 ▶).

## Supplementary Material

Crystal structure: contains datablocks global, I. DOI: 10.1107/S1600536809050971/hb5254sup1.cif

Structure factors: contains datablocks I. DOI: 10.1107/S1600536809050971/hb5254Isup2.hkl

Additional supplementary materials:  crystallographic information; 3D view; checkCIF report

## Figures and Tables

**Table 1:** Hydrogen-bond geometry (Å, °)

*D*—H⋯*A*	*D*—H	H⋯*A*	*D*⋯*A*	*D*—H⋯*A*
N2—H1*N*2⋯O2^i^	0.87 (3)	1.96 (3)	2.790 (2)	159 (3)
O1—H1*O*1⋯N1	0.90 (4)	1.83 (4)	2.626 (3)	147 (4)
C14—H14*A*⋯O1^i^	0.93	2.51	3.272 (4)	139
C20—H20*C*⋯*Cg*1^ii^	0.96	2.80	3.722 (3)	161

Symmetry codes: (i) 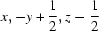; (ii) 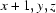. *Cg*1 is the centroid of the C10–C15 benzene ring.

**Fig. 1.**
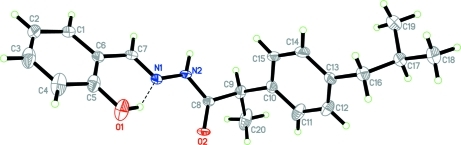
The molecular structure of (I), showing 50% probability displacement ellipsoids for non-H atoms. The intramolecular hydrogen bond is shown as a dashed line.

**Fig. 2.**
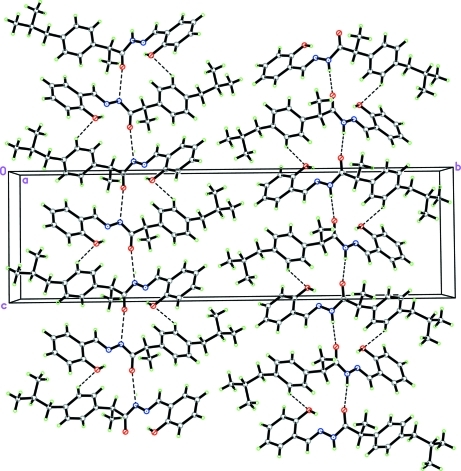
The crystal structure of (I), viewed along the a axis, showing one-dimensional extended chains of R22(16) ring motif along the c axis. Intermolecular hydrogen bonds are shown as dashed lines.

## supplementary crystallographic information

### Comment

Hydrazone compounds are obtained by the reaction of aromatic and heterocyclic hydrazides with mono- and di-aldehydes or ketones and they have revealed very versatile behaviour in metal coordination (Rodrìguez-Argüelles *et al.*, 2004). Hydrazones have been demonstrated to possess a variety of pharmacological activities (Bedia *et al.*, 2006; Rollas *et al.*, 2002; Terzioglu & Gürsoy, 2003). These observations have been the guidelines for the development of new hydrazone incorporating ibuprofen. Prompted by these observations and in continuation of our work (Fun *et al.*, 2009*b*), we herein report the structure of this new hydrazone.

The title hydrazide compound (Fig. 1) exists in a *trans* configuration with respect to the acyclic C7═N1 bond. An intramolecular O1—H1O1···N1 hydrogen bond (Table 1) generates a six-membered ring, producing an *S*(6) ring motif (Bernstein *et al.*, 1995). The mean plane through the formohydrazide unit (N1/N2/C8/O2) is essentially planar, with maximum deviation of 0.025 (2) Å for atom C8. This plane is inclined to the C1-C6 benzene ring at a dihedral angle of 24.45 (16)°, and almost perpendicular to the C10-C15 benzene ring, as indicated by the dihedral angle of 87.14 (16)°. The bond lengths and angles are within normal ranges and comparable to those closely related structures (Fun *et al.*, 2009*a,b*).

In the crystal packing (Fig. 2), intermolecular N2—H1N2···O2 and C14—H14A···O1 hydrogen bonds (Table 1) link neighbouring molecules into one-dimensional infinite chains of *R*^2^_2_(16) hydrogen bond ring motifs (Bernstein *et al.*, 1995) along the *c* axis. An intermolecular C—H···π interaction (Table 1) involving the C10-C15 benzene ring is also observed.

### Experimental

The title compound was obtained by refluxing for 1 h salicylaldehyde (0.01 mol), 2-(4-isobutylphenyl)propanehydrazide (0.01 mol) and ethanol (30 ml) with the addition of three drops of concentrated sulphuric acid. The solid product obtained was filtered, washed with ethanol and dried. Colourless blocks of (I) were obtained by slow evaporation from ethanol. Yield was 74 %. *M.p.* 426 K.

### Refinement

The H atoms bound to atoms O1 and N2 were located from the difference Fourier map and allowed to refine freely. All the other H atoms were placed in calculated positions, with C—H = 0.93 – 0.97 Å, and as riding, with *U*_iso_ = 1.2 or 1.5 *U*_eq_(C). A rotating group model was used for the methyl groups. The reflection (020) was omitted from the refinement as the intensity was affected by the beam backstop.

### Crystal data

**Table:**

C_20_H_24_N_2_O_2_	*F*(000) = 696
*M**_r_* = 324.41	*D*_x_ = 1.220 Mg m^−^^3^
Monoclinic, *P*2_1_/*c*	Mo *K*α radiation, λ = 0.71073 Å
Hall symbol: -P 2ybc	Cell parameters from 7778 reflections
*a* = 5.5017 (2) Å	θ = 2.4–30.0°
*b* = 33.0204 (14) Å	µ = 0.08 mm^−^^1^
*c* = 9.7279 (4) Å	*T* = 100 K
β = 91.731 (3)°	Block, colourless
*V* = 1766.45 (12) Å^3^	0.39 × 0.24 × 0.19 mm
*Z* = 4	

### Data collection

**Table:**

Bruker SMART APEXII CCD diffractometer	4059 independent reflections
Radiation source: fine-focus sealed tube	3190 reflections with *I* > 2σ(*I*)
graphite	*R*_int_ = 0.042
φ and ω scans	θ_max_ = 27.5°, θ_min_ = 2.2°
Absorption correction: multi-scan (*SADABS*; Bruker, 2005)	*h* = −7→7
*T*_min_ = 0.970, *T*_max_ = 0.985	*k* = −38→42
18817 measured reflections	*l* = −12→12

### Refinement

**Table:**

Refinement on *F*^2^	Primary atom site location: structure-invariant direct methods
Least-squares matrix: full	Secondary atom site location: difference Fourier map
*R*[*F*^2^ > 2σ(*F*^2^)] = 0.076	Hydrogen site location: inferred from neighbouring sites
*wR*(*F*^2^) = 0.181	H atoms treated by a mixture of independent and constrained refinement
*S* = 1.18	*w* = 1/[σ^2^(*F*_o_^2^) + (0.0443*P*)^2^ + 2.5698*P*] where *P* = (*F*_o_^2^ + 2*F*_c_^2^)/3
4059 reflections	(Δ/σ)_max_ < 0.001
228 parameters	Δρ_max_ = 0.26 e Å^−^^3^
0 restraints	Δρ_min_ = −0.28 e Å^−^^3^

### Special details

**Table:**

Experimental. The crystal was placed in the cold stream of an Oxford Cyrosystems Cobra open-flow nitrogen cryostat (Cosier & Glazer, 1986) operating at 100.0 (1)K.
Geometry. All esds (except the esd in the dihedral angle between two l.s. planes) are estimated using the full covariance matrix. The cell esds are taken into account individually in the estimation of esds in distances, angles and torsion angles; correlations between esds in cell parameters are only used when they are defined by crystal symmetry. An approximate (isotropic) treatment of cell esds is used for estimating esds involving l.s. planes.
Refinement. Refinement of F^2^ against ALL reflections. The weighted R-factor wR and goodness of fit S are based on F^2^, conventional R-factors R are based on F, with F set to zero for negative F^2^. The threshold expression of F^2^ > 2sigma(F^2^) is used only for calculating R-factors(gt) etc. and is not relevant to the choice of reflections for refinement. R-factors based on F^2^ are statistically about twice as large as those based on F, and R- factors based on ALL data will be even larger.

### Fractional atomic coordinates and isotropic or equivalent isotropic displacement parameters (Å^2^)

**Table:**

	*x*	*y*	*z*	*U*_iso_*/*U*_eq_	
O1	−0.2604 (4)	0.18426 (6)	0.5584 (2)	0.0358 (5)	
O2	0.3223 (3)	0.25847 (5)	0.60286 (15)	0.0209 (4)	
N1	0.1237 (4)	0.20555 (6)	0.4218 (2)	0.0199 (5)	
N2	0.3012 (4)	0.23282 (6)	0.3875 (2)	0.0210 (5)	
C1	−0.1353 (6)	0.11240 (9)	0.2812 (3)	0.0364 (7)	
H1A	−0.0286	0.1087	0.2099	0.044*	
C2	−0.3138 (6)	0.08401 (9)	0.3013 (3)	0.0367 (8)	
H2A	−0.3268	0.0614	0.2448	0.044*	
C3	−0.4735 (6)	0.08962 (9)	0.4065 (4)	0.0385 (8)	
H3A	−0.5952	0.0706	0.4205	0.046*	
C4	−0.4540 (5)	0.12339 (9)	0.4918 (4)	0.0382 (7)	
H4A	−0.5625	0.1268	0.5624	0.046*	
C5	−0.2736 (5)	0.15194 (8)	0.4721 (3)	0.0285 (6)	
C6	−0.1095 (5)	0.14680 (8)	0.3650 (3)	0.0269 (6)	
C7	0.0819 (5)	0.17603 (8)	0.3390 (3)	0.0276 (6)	
H7A	0.1748	0.1732	0.2613	0.033*	
C8	0.3850 (4)	0.25968 (7)	0.4827 (2)	0.0156 (5)	
C9	0.5550 (5)	0.29208 (8)	0.4303 (3)	0.0235 (5)	
H9A	0.6170	0.2834	0.3417	0.028*	
C10	0.4016 (5)	0.33029 (8)	0.4081 (3)	0.0235 (5)	
C11	0.4132 (5)	0.36299 (8)	0.4983 (3)	0.0310 (6)	
H11A	0.5260	0.3628	0.5715	0.037*	
C12	0.2587 (5)	0.39598 (8)	0.4807 (3)	0.0277 (6)	
H12A	0.2735	0.4177	0.5411	0.033*	
C13	0.0842 (6)	0.39741 (8)	0.3763 (3)	0.0277 (6)	
C14	0.0756 (8)	0.36506 (9)	0.2855 (3)	0.0518 (11)	
H14A	−0.0389	0.3652	0.2131	0.062*	
C15	0.2335 (7)	0.33241 (9)	0.2998 (3)	0.0438 (9)	
H15A	0.2259	0.3116	0.2354	0.053*	
C16	−0.0929 (6)	0.43214 (8)	0.3616 (3)	0.0319 (7)	
H16A	−0.1484	0.4392	0.4522	0.038*	
H16B	−0.2334	0.4230	0.3075	0.038*	
C17	0.0063 (6)	0.47049 (8)	0.2944 (3)	0.0331 (7)	
H17A	0.1393	0.4809	0.3537	0.040*	
C18	−0.1918 (7)	0.50278 (9)	0.2851 (3)	0.0435 (8)	
H18A	−0.1264	0.5272	0.2472	0.065*	
H18B	−0.2499	0.5082	0.3754	0.065*	
H18C	−0.3239	0.4933	0.2270	0.065*	
C19	0.1076 (7)	0.46188 (9)	0.1530 (3)	0.0402 (8)	
H19A	0.1558	0.4869	0.1114	0.060*	
H19B	−0.0152	0.4491	0.0960	0.060*	
H19C	0.2460	0.4443	0.1628	0.060*	
C20	0.7674 (5)	0.29712 (9)	0.5318 (4)	0.0423 (8)	
H20A	0.8636	0.2729	0.5335	0.063*	
H20B	0.7074	0.3022	0.6218	0.063*	
H20C	0.8658	0.3195	0.5043	0.063*	
H1N2	0.346 (6)	0.2342 (9)	0.303 (3)	0.034 (9)*	
H1O1	−0.127 (8)	0.1984 (12)	0.539 (4)	0.064 (13)*	

### Atomic displacement parameters (Å^2^)

**Table:**

	*U*^11^	*U*^22^	*U*^33^	*U*^12^	*U*^13^	*U*^23^
O1	0.0260 (11)	0.0248 (10)	0.0569 (14)	−0.0014 (8)	0.0068 (10)	−0.0075 (9)
O2	0.0300 (10)	0.0253 (9)	0.0074 (7)	0.0012 (7)	0.0012 (7)	−0.0001 (7)
N1	0.0248 (11)	0.0194 (10)	0.0150 (9)	−0.0027 (8)	−0.0055 (8)	0.0027 (8)
N2	0.0329 (12)	0.0221 (11)	0.0083 (9)	−0.0060 (9)	0.0021 (8)	0.0006 (8)
C1	0.060 (2)	0.0304 (15)	0.0184 (13)	−0.0137 (14)	−0.0084 (13)	0.0022 (11)
C2	0.057 (2)	0.0258 (15)	0.0268 (14)	−0.0115 (13)	−0.0136 (14)	0.0030 (12)
C3	0.0319 (16)	0.0256 (15)	0.057 (2)	−0.0059 (12)	−0.0153 (15)	0.0061 (14)
C4	0.0206 (14)	0.0292 (15)	0.065 (2)	0.0004 (11)	−0.0005 (14)	−0.0014 (14)
C5	0.0243 (13)	0.0198 (13)	0.0409 (16)	0.0032 (10)	−0.0087 (12)	0.0015 (11)
C6	0.0379 (15)	0.0226 (13)	0.0192 (12)	−0.0055 (11)	−0.0128 (11)	0.0059 (10)
C7	0.0434 (16)	0.0256 (14)	0.0134 (11)	−0.0049 (12)	−0.0048 (11)	0.0025 (10)
C8	0.0146 (11)	0.0190 (11)	0.0132 (10)	0.0031 (9)	−0.0001 (8)	0.0004 (9)
C9	0.0239 (13)	0.0202 (12)	0.0272 (13)	−0.0009 (10)	0.0118 (10)	−0.0033 (10)
C10	0.0311 (14)	0.0191 (12)	0.0212 (12)	−0.0024 (10)	0.0130 (10)	0.0016 (10)
C11	0.0232 (14)	0.0261 (14)	0.0435 (16)	−0.0025 (11)	−0.0013 (12)	−0.0117 (12)
C12	0.0255 (14)	0.0239 (13)	0.0339 (15)	−0.0010 (11)	0.0057 (11)	−0.0101 (11)
C13	0.0454 (17)	0.0201 (13)	0.0181 (12)	0.0021 (11)	0.0076 (11)	0.0042 (10)
C14	0.105 (3)	0.0295 (16)	0.0196 (14)	0.0204 (18)	−0.0219 (17)	−0.0017 (12)
C15	0.095 (3)	0.0228 (14)	0.0131 (12)	0.0159 (16)	−0.0038 (15)	−0.0027 (11)
C16	0.0447 (18)	0.0244 (14)	0.0265 (14)	0.0061 (12)	0.0002 (12)	0.0016 (11)
C17	0.0498 (19)	0.0229 (14)	0.0262 (14)	0.0030 (12)	−0.0069 (13)	0.0019 (11)
C18	0.062 (2)	0.0271 (15)	0.0413 (18)	0.0081 (15)	−0.0068 (16)	0.0036 (13)
C19	0.057 (2)	0.0328 (16)	0.0305 (16)	0.0008 (14)	−0.0011 (14)	0.0074 (13)
C20	0.0220 (14)	0.0266 (15)	0.078 (2)	−0.0012 (11)	−0.0006 (15)	−0.0134 (15)

### Geometric parameters (Å, °)

**Table:**

O1—C5	1.359 (3)	C11—C12	1.389 (4)
O1—H1O1	0.89 (4)	C11—H11A	0.9300
O2—C8	1.229 (3)	C12—C13	1.377 (4)
N1—C7	1.281 (3)	C12—H12A	0.9300
N1—N2	1.377 (3)	C13—C14	1.386 (4)
N2—C8	1.354 (3)	C13—C16	1.509 (4)
N2—H1N2	0.86 (3)	C14—C15	1.389 (4)
C1—C2	1.376 (4)	C14—H14A	0.9300
C1—C6	1.403 (4)	C15—H15A	0.9300
C1—H1A	0.9300	C16—C17	1.533 (4)
C2—C3	1.382 (5)	C16—H16A	0.9700
C2—H2A	0.9300	C16—H16B	0.9700
C3—C4	1.392 (4)	C17—C18	1.526 (4)
C3—H3A	0.9300	C17—C19	1.526 (4)
C4—C5	1.387 (4)	C17—H17A	0.9800
C4—H4A	0.9300	C18—H18A	0.9600
C5—C6	1.409 (4)	C18—H18B	0.9600
C6—C7	1.456 (4)	C18—H18C	0.9600
C7—H7A	0.9300	C19—H19A	0.9600
C8—C9	1.519 (3)	C19—H19B	0.9600
C9—C20	1.516 (4)	C19—H19C	0.9600
C9—C10	1.530 (4)	C20—H20A	0.9600
C9—H9A	0.9800	C20—H20B	0.9600
C10—C15	1.382 (4)	C20—H20C	0.9600
C10—C11	1.392 (4)		
			
C5—O1—H1O1	108 (3)	C13—C12—H12A	119.1
C7—N1—N2	117.4 (2)	C11—C12—H12A	119.1
C8—N2—N1	119.4 (2)	C12—C13—C14	117.0 (3)
C8—N2—H1N2	121 (2)	C12—C13—C16	122.0 (2)
N1—N2—H1N2	119 (2)	C14—C13—C16	121.0 (3)
C2—C1—C6	122.0 (3)	C13—C14—C15	121.6 (3)
C2—C1—H1A	119.0	C13—C14—H14A	119.2
C6—C1—H1A	119.0	C15—C14—H14A	119.2
C1—C2—C3	119.1 (3)	C10—C15—C14	121.2 (3)
C1—C2—H2A	120.4	C10—C15—H15A	119.4
C3—C2—H2A	120.4	C14—C15—H15A	119.4
C2—C3—C4	120.6 (3)	C13—C16—C17	115.5 (3)
C2—C3—H3A	119.7	C13—C16—H16A	108.4
C4—C3—H3A	119.7	C17—C16—H16A	108.4
C5—C4—C3	120.3 (3)	C13—C16—H16B	108.4
C5—C4—H4A	119.8	C17—C16—H16B	108.4
C3—C4—H4A	119.8	H16A—C16—H16B	107.5
O1—C5—C4	118.3 (3)	C18—C17—C19	110.9 (2)
O1—C5—C6	121.9 (2)	C18—C17—C16	109.8 (3)
C4—C5—C6	119.8 (3)	C19—C17—C16	112.0 (2)
C1—C6—C5	118.1 (3)	C18—C17—H17A	108.0
C1—C6—C7	119.8 (3)	C19—C17—H17A	108.0
C5—C6—C7	122.1 (2)	C16—C17—H17A	108.0
N1—C7—C6	120.8 (3)	C17—C18—H18A	109.5
N1—C7—H7A	119.6	C17—C18—H18B	109.5
C6—C7—H7A	119.6	H18A—C18—H18B	109.5
O2—C8—N2	122.0 (2)	C17—C18—H18C	109.5
O2—C8—C9	122.4 (2)	H18A—C18—H18C	109.5
N2—C8—C9	115.6 (2)	H18B—C18—H18C	109.5
C20—C9—C8	109.2 (2)	C17—C19—H19A	109.5
C20—C9—C10	114.4 (2)	C17—C19—H19B	109.5
C8—C9—C10	106.6 (2)	H19A—C19—H19B	109.5
C20—C9—H9A	108.8	C17—C19—H19C	109.5
C8—C9—H9A	108.8	H19A—C19—H19C	109.5
C10—C9—H9A	108.8	H19B—C19—H19C	109.5
C15—C10—C11	117.3 (3)	C9—C20—H20A	109.5
C15—C10—C9	120.2 (2)	C9—C20—H20B	109.5
C11—C10—C9	122.4 (2)	H20A—C20—H20B	109.5
C12—C11—C10	120.9 (3)	C9—C20—H20C	109.5
C12—C11—H11A	119.5	H20A—C20—H20C	109.5
C10—C11—H11A	119.5	H20B—C20—H20C	109.5
C13—C12—C11	121.9 (3)		
			
C7—N1—N2—C8	−167.2 (2)	N2—C8—C9—C10	99.7 (2)
C6—C1—C2—C3	0.4 (4)	C20—C9—C10—C15	167.3 (3)
C1—C2—C3—C4	−0.3 (4)	C8—C9—C10—C15	−71.9 (3)
C2—C3—C4—C5	0.0 (4)	C20—C9—C10—C11	−16.6 (4)
C3—C4—C5—O1	−179.5 (3)	C8—C9—C10—C11	104.2 (3)
C3—C4—C5—C6	0.1 (4)	C15—C10—C11—C12	1.0 (4)
C2—C1—C6—C5	−0.2 (4)	C9—C10—C11—C12	−175.2 (3)
C2—C1—C6—C7	−179.7 (3)	C10—C11—C12—C13	1.7 (4)
O1—C5—C6—C1	179.6 (2)	C11—C12—C13—C14	−2.5 (5)
C4—C5—C6—C1	0.0 (4)	C11—C12—C13—C16	176.6 (3)
O1—C5—C6—C7	−0.9 (4)	C12—C13—C14—C15	0.7 (5)
C4—C5—C6—C7	179.4 (3)	C16—C13—C14—C15	−178.4 (3)
N2—N1—C7—C6	−177.5 (2)	C11—C10—C15—C14	−2.8 (5)
C1—C6—C7—N1	−173.3 (2)	C9—C10—C15—C14	173.5 (3)
C5—C6—C7—N1	7.3 (4)	C13—C14—C15—C10	2.0 (6)
N1—N2—C8—O2	6.1 (3)	C12—C13—C16—C17	79.8 (3)
N1—N2—C8—C9	−172.1 (2)	C14—C13—C16—C17	−101.1 (3)
O2—C8—C9—C20	45.5 (3)	C13—C16—C17—C18	179.5 (2)
N2—C8—C9—C20	−136.3 (2)	C13—C16—C17—C19	55.8 (3)
O2—C8—C9—C10	−78.5 (3)		

### Hydrogen-bond geometry (Å, °)

**Table:**

*D*—H···*A*	*D*—H	H···*A*	*D*···*A*	*D*—H···*A*
N2—H1N2···O2^i^	0.87 (3)	1.96 (3)	2.790 (2)	159 (3)
O1—H1O1···N1	0.90 (4)	1.83 (4)	2.626 (3)	147 (4)
C14—H14A···O1^i^	0.93	2.51	3.272 (4)	139
C20—H20C···Cg1^ii^	0.96	2.80	3.722 (3)	161

Symmetry codes: (i) *x*, −*y*+1/2, *z*−1/2; (ii) *x*+1, *y*, *z*.
